# Repurposing Semaglutide and Liraglutide for Alcohol Use Disorder

**DOI:** 10.1001/jamapsychiatry.2024.3599

**Published:** 2024-11-13

**Authors:** Markku Lähteenvuo, Jari Tiihonen, Anssi Solismaa, Antti Tanskanen, Ellenor Mittendorfer-Rutz, Heidi Taipale

**Affiliations:** 1 Department of Forensic Psychiatry, University of Eastern Finland, Niuvanniemi Hospital, Kuopio, Finland; 2 Department of Clinical Neuroscience, Karolinska Institutet, Stockholm, Sweden; 3 Center for Psychiatry Research, Stockholm City Council, Stockholm, Sweden; 4 Department of Psychiatry, Faculty of Medicine and Health Technology, Tampere University, Tampere, Finland; 5 Department of Psychiatry, The Pirkanmaa Wellbeing Services County, Tampere, Finland; 6 School of Pharmacy, University of Eastern Finland, Kuopio, Finland

## Abstract

**Importance:**

Preliminary studies suggest that glucagon-like peptide-1 receptor (GLP-1) agonists, used to treat type 2 diabetes and obesity, may decrease alcohol consumption.

**Objective:**

To test whether the risk of hospitalization due to alcohol use disorder (AUD) is decreased during the use of GLP-1 agonists compared with periods of nonuse for the same individual.

**Design, Setting, and Participants:**

This cohort study was an observational study conducted nationwide in Sweden using data from January 2006 to December 2023. The population-based cohort was identified from registers of inpatient care, specialized outpatient care, sickness absence, and disability pension. Participants were all residents aged 16 to 64 years who had a diagnosis of AUD.

**Exposures:**

The primary exposure was use of individual GLP-1 agonists (compared with nonuse of GLP-1 agonists), and the secondary exposure was medications with indication for AUD.

**Main Outcomes and Measures:**

The primary outcome was AUD hospitalization analyzed in a Cox regression within-individual model. Secondary outcomes were any substance use disorder (SUD)–related hospitalization, somatic hospitalization, and suicide attempt.

**Results:**

The cohort included 227 866 individuals with AUD; 144 714 (63.5%) were male and 83 154 (36.5%) were female, with a mean (SD) age of 40.0 (15.7) years. Median (IQR) follow-up time was 8.8 (4.0-13.3) years. A total of 133 210 individuals (58.5%) experienced AUD hospitalization. Semaglutide (4321 users) was associated with the lowest risk (AUD: adjusted hazard ratio [aHR], 0.64; 95% CI, 0.50-0.83; any SUD: aHR, 0.68; 95% CI, 0.54-0.85) and use of liraglutide (2509 users) with the second lowest risk (AUD: aHR, 0.72; 95% CI, 0.57-0.92; any SUD: aHR, 0.78; 95% CI, 0.64-0.97) of both AUD and SUD hospitalization. Use of any AUD medication was associated with a modestly decreased risk (aHR, 0.98; 95% CI, 0.96-1.00). Semaglutide (aHR, 0.78; 95% CI, 0.68-0.90) and liraglutide (aHR, 0.79; 95% CI, 0.69-0.91) use were also associated with decreased risk of somatic hospitalizations but not associated with suicide attempts (semaglutide: aHR, 0.55; 95% CI, 0.23-1.30; liraglutide: aHR, 1.08; 95% CI, 0.55-2.15).

**Conclusions and Relevance:**

Among patients with AUD and comorbid obesity/type 2 diabetes, the use of semaglutide and liraglutide were associated with a substantially decreased risk of hospitalization due to AUD. This risk was lower than that of officially approved AUD medications. Semaglutide and liraglutide may be effective in the treatment of AUD, and clinical trials are urgently needed to confirm these findings.

## Key Points

**Question:**

Are glucagon-like peptide-1 receptor (GLP-1) agonists effective in the treatment of alcohol use disorder?

**Findings:**

This cohort study with a median follow-up time of more than 8 years indicates that individuals are at markedly lower risk of alcohol-related hospitalizations and hospitalizations due to somatic reasons when using GLP-1 agonists, especially semaglutide, as compared with times they are not using them.

**Meaning:**

GLP-1 agonists, especially semaglutide, offer promise as a novel treatment to reduce alcohol consumption and to prevent development of alcohol-related outcomes, but randomized clinical trials are needed to verify these initial findings.

## Teaser

This cohort study examines nationwide registry data for Swedish residents with a diagnosis of alcohol use disorder to determine whether hospitalization risk for an individual is decreased during periods of GLP-1 agonist use vs periods of nonuse.

## Introduction

According to the World Health Organization, harmful use of alcohol is accountable for 5.1% of the global burden of disease.^1^ Psychosocial treatments are the cornerstone of alcohol use disorder (AUD) treatment, but pharmacological treatments are also beneficial, although underused.^2,3^

Glucagon-like peptide-1 receptor (GLP-1) agonists are approved for clinical use to treat diabetes and obesity. Preclinical studies in rodents and monkeys, as well as human case reports, have shown that GLP-1 agonists can reduce alcohol consumption.^4,5,6^ In humans, genetic variation in *GLP-1R* has been shown to be associated with increased risk of AUD.^7^ A recent registry study from Denmark has also shown that use of GLP-1 agonists has been linked to transient (3-month) reduced risk of subsequent alcohol-related events.^8^

We aimed to investigate the potential of GLP-1 agonists as a treatment for reducing alcohol-related harms by analyzing real-world data from Swedish registries.

## Methods

### Study Design and Cohort Acquisition

Swedish nationwide electronic registries were used to obtain and combine data through personal deidentified identification numbers. The project was approved by the Regional Ethical Review Board, Karolinska Institutet, Stockholm, Sweden (Dnr: 2007/762-31 and Dnr: 2021-06441-02). Informed consent is not required in Sweden for register-based studies, where no contact is made with the patient.

The National Patient registry (inpatient and specialized outpatient visits) and Microdata for Analysis of Social Insurance register (data on sickness absence and disability pension diagnoses) were used to identify individuals diagnosed with AUD (*International Statistical Classification of Diseases and Related Health Problems, Tenth Revision* [*ICD-10*], code F10) during the years 2006-2021 who were age 16 to 65 years. This produced a cohort of 227 886 individuals who were followed up from AUD diagnosis to death, emigration, or end of data linkage (December 31, 2023), whichever came first.

### Exposure

Individual drug use periods were constructed using the PRE2DUP method with data from the National Prescribed Drug Register.^9,10^ The main exposure was GLP-1 agonists, which were exenatide, liraglutide, dulaglutide, and semaglutide; lixisenatide was excluded because of sparsity of use. The secondary exposure was use of AUD medications (disulfiram, acamprosate, and naltrexone; nalmefene excluded because of sparsity of use). A group category including all the previously mentioned AUD medications was also constructed.

### Outcomes

The main outcome measure was hospitalization due to AUD (*ICD-10* F10). Secondary outcomes were hospitalization due to substance use disorder (SUD) (F10-F16, F18-F19), hospitalization due to somatic reasons (A00-N99, U07[ = COVID-19], but excluding F00-F99), and hospitalization due to suicide attempt (X60-X84, Y10-Y34).

### Statistical Analysis

We used a within-individual design where each individual acts as their own control. Cox regression models with fixed effects were used to calculate the within-individual risk of an outcome associated with use vs nonuse of pharmacotherapies (eFigures 1 and 2 in Supplement 1).^11^ The use of individual GLP-1 agonists was compared with nonuse of GLP-1 agonists. Groupwise and use of specific AUD medications were compared with nonuse of AUD medications.

Results are presented as adjusted hazard ratios (aHRs) and 95% CIs. Exposures with fewer than 10 events were excluded from the figures (exenatide for AUD and suicide attempt analyses). Data analyses were conducted from April to September 2024 and used SAS version 9.4 (SAS Institute).

## Results

The total cohort consisted of 227 868 individuals with an AUD, of whom 144 714 (63.5%) were male and 83 154 (36.5%) female (Table). Their mean (SD) age was 40.0 (15.7) years at cohort entry, and 193 719 (85%) were born in Sweden. The cohort included 6276 individuals with both an AUD and use of GLP-1 agonists. Of this subcohort, 4058 (64.7%) were male and 2218 (35.3) female; the mean (SD) age for the subcohort was 46.0 (12.5) years. The cohort was followed up for a median (IQR) of 8.8 (4.0-13.3) years.

**Table.** Characteristics of the Whole Cohort and Subcohorts of Patients Who Used GLP-1 Agonists and Those Who Used AUD Medications During Follow-Up^a^

Characteristic	No. (%)		
	All patients (N = 227 868)	GLP-1 agonist users (n = 6276)	AUD medication users (n = 75 454)
Age, mean (SD), y	40.0 (15.7)	46.0 (12.5)	45.4 (12.4)
Sex			
Male	144 714 (63.5)	4058 (64.7)	50 246 (66.6)
Female	83 154 (36.5)	2218 (35.3)	25 208 (33.4)
Country of birth			
Sweden	193 719 (85.0)	5131 (81.8)	65 893 (87.3)
Other European country	20 721 (9.1)	640 (10.2)	6946 (9.2)
Outside Europe	13 426 (5.9)	505 (8.1)	2615 (3.5)
Sickness absence during previous year before cohort entry			
0 d	184 070 (80.8)	4561 (72.7)	54 417 (72.1)
1-90 d	28 239 (12.4)	1032 (16.4)	13 659 (18.1)
>90 d	15 557 (6.8)	683 (10.9)	7378 (9.8)
Disability pension	32 818 (14.4)	1162 (18.5)	11 437 (15.2)
Type of AUD diagnosis at baseline^b^			
Acute intoxication (F10.0)	90 225 (39.6)	1951 (31.1)	10 973 (14.5)
Dependence syndrome (F10.2)	63 849 (28.0)	2131 (34.0)	34 160 (45.3)
Harmful use (F10.1)	49 100 (21.6)	1539 (24.5)	20 435 (27.1)
Other/unspecified	203 174 (10.8)	655 (10.4)	9886 (13.1)
Other substance use disorder before baseline	8899 (3.9)	285 (4.5)	3198 (4.2)
Suicide attempt before baseline	20 349 (8.9)	794 (12.7)	7460 (9.9)
Diseases recorded by end of follow-up			
Type 2 diabetes	14 787 (6.5)	2745 (43.7)	5999 (8.0)
Cardiovascular disease	60 251 (26.4)	3178 (50.6)	25 176 (33.4)
Kidney disease	13 634 (6.0)	671 (10.7)	5094 (6.8)
Obesity	10 818 (4.8)	1175 (18.7)	4998 (6.6)

Abbreviations: AUD, alcohol use disorder; GLP-1, glucagon-like peptide-1 receptor.

^a^ Before cohort entry/before baseline data recorded from 1997 onwards. Sociodemographic variables were derived from the longitudinal integrated database for health insurance and labor market studies register.

^b^ Parentheses include codes from *International Statistical Classification of Diseases and Related Health Problems, Tenth Revision.*

### Risk of Hospitalization Due to AUD and SUD

**Figure 1.**
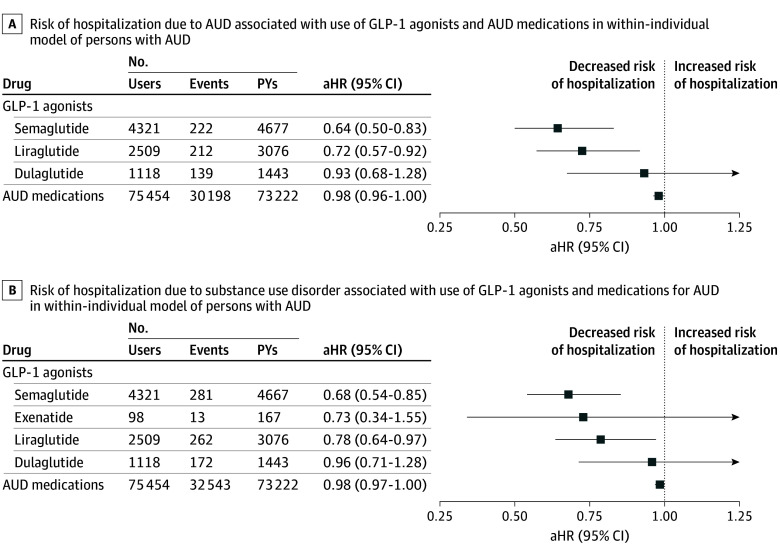
Risk of Hospitalization Due to Alcohol Use Disorder (AUD) and Substance Use Disorder (SUD) The use of individual glucagon-like peptide-1 receptor (GLP-1) agonists was compared with nonuse of GLP-1 agonists. Groupwise and use of specific AUD medications were compared with nonuse of AUD medications. Both of these models (A and B) were adjusted for time-varying use of psychotropic medications (antipsychotics, N05A; antidepressants, N06A; mood stabilizers, including carbamazepine, N03AF01; valproic acid, N03AG01; lamotrigine, N03AX09; and lithium, N05AN01), benzodiazepines and related drugs (N05BA, N05CD, N05CF), and attention-deficit/hyperactivity disorder (ADHD) medications (N06BA); use of antidiabetic drugs other than GLP-1 agonists (A10 excluding A10BJ); temporal order of GLP-1 medication; and time since cohort entry. aHR indicates adjusted hazard ratio.

A total of 133 210 individuals were hospitalized because of AUD and 138 390 because of any SUD at least once. Use of semaglutide was associated with the lowest risk (AUD: aHR, 0.64; 95% CI, 0.50-0.83; any SUD: aHR, 0.68, 95% CI, 0.54-0.85) and use of liraglutide with the second lowest risk (AUD: aHR, 0.72; 95% CI, 0.57-0.92; any SUD: aHR, 0.78; 95% CI, 0.64-0.97) of both AUD and SUD hospitalization (Figure 1). Use of AUD medications in general was not associated with significantly altered risk of either AUD or SUD hospitalization (AUD: aHR, 0.98; 95% CI, 0.96-1.00; any SUD: aHR, 0.98; 95% CI, 0.97-1.00), although use of naltrexone was associated with reduced risk (AUD: aHR, 0.86; 95% CI, 0.83-0.89; any SUD: aHR, 0.86; 95% CI, 0.84-0.90). The results for individual AUD medications are shown in eTable 1 in Supplement 1. Additional sensitivity analyses to assess bias are presented in eTables 2 and 3 in Supplement 1.

### Risk of Hospitalization Due to Somatic Reasons and Suicide Attempts

**Figure 2.**
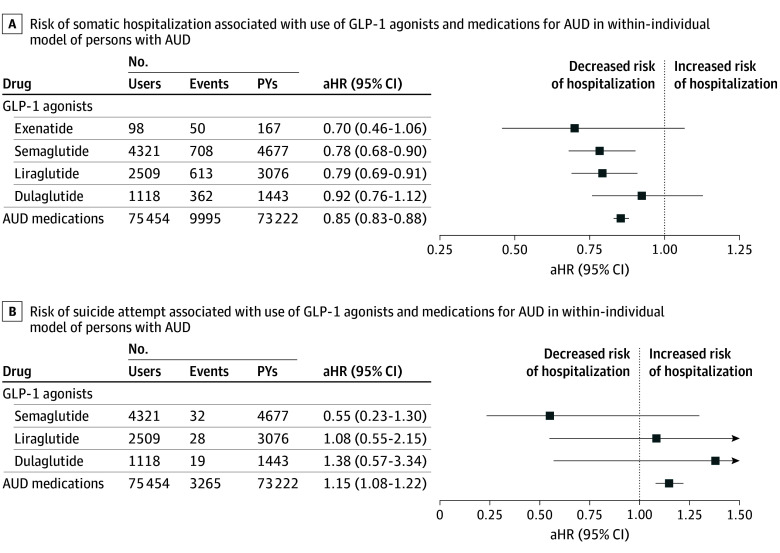
Risk of Hospitalization Due to Somatic Reasons and Suicide Attempt The use of individual glucagon-like peptide-1 receptor (GLP-1) agonists was compared with nonuse of GLP-1 agonists. Groupwise and use of specific alcohol use disorder (AUD) medications were compared with nonuse of AUD medications. Both of these models (A and B) were adjusted for time-varying use of psychotropic medications (antipsychotics, N05A; antidepressants, N06A; mood stabilizers, including carbamazepine, N03AF01; valproic acid, N03AG01; lamotrigine, N03AX09; and lithium, N05AN01), benzodiazepines and related drugs (N05BA, N05CD, N05CF), and attention-deficit/hyperactivity disorder medications (N06BA); use of antidiabetic drugs other than GLP-1 agonists (A10 excluding A10BJ); temporal order of GLP-1 medication; and time since cohort entry. aHR indicates adjusted hazard ratio.

A total of 83 166 individuals were hospitalized for somatic reasons and 22 231 for suicide attempt. Use of semaglutide was associated with the lowest risk (aHR, 0.78; 95% CI, 0.68-0.90) and use of liraglutide with the second lowest risk (aHR, 0.79; 95% CI, 0.69-0.91) of somatic hospitalization (Figure 2). Use of AUD medications in general was associated with reduced risk of somatic hospitalization (aHR, 0.85; 95% CI, 0.83-0.88).

Use of GLP-1 agonists was not associated with a statistically significantly altered risk of suicide attempt (semaglutide: aHR, 0.55, 95% CI, 0.23-1.30). However, use of AUD medications in general was associated with an increased risk of suicide attempt (aHR, 1.15, 95% CI, 1.08-1.22). The results for individual AUD medications are shown in eTable 1 in Supplement 1.

## Discussion

In this nationwide register-based study, the GLP-1 agonists semaglutide and liraglutide, but not other GLP-1 agonists, were associated with a markedly reduced risk of AUD- and SUD-related hospitalizations as well as somatic hospitalizations. We did not observe statistically significant changes in risk of suicide attempts for GLP-1 agonists, although the point estimate of 0.55 for semaglutide suggests it may be associated with decreased risk of suicide. Especially interesting is the notion that semaglutide and liraglutide were associated with better outcomes than AUD medications (naltrexone, disulfiram, and acamprosate), although this comparison needs to be taken with a grain of salt, as the comparators were different: GLP-1 agonist use was compared with the times GLP-1 agonists were not used, and AUD medication use compared with times when AUD medications were not used.

The result for SUD-related hospitalizations needs to be interpreted cautiously, as the majority of these hospitalizations were from alcohol-related causes. However, this result is in line with a recent registry study, which found that use of semaglutide was associated with reduced incidence and relapse of cannabis use disorder.^12^ As the GLP-1 receptor has been shown to be involved in many pathways related to craving and reward,^13^ it may be plausible that GLP-1 agonists could be used for a wide variety of addictions. A recent review suggested that GLP-1 agonists may exert a centrally mediated effect to reduce addictive behavior at least partly via dopamine modulation.^14^

### Limitations

Because this is an observational study, it can only speak for associations, not causality. Further strengths and weaknesses are discussed in the eAppendix in Supplement 1.

## Conclusions

AUDs and SUDs are undertreated pharmacologically, despite the availability of effective treatments. However, novel treatments are also needed because existing treatments may not be suitable for all patients. GLP-1 agonists, and especially semaglutide and liraglutide, may be effective in the treatment of AUD. Randomized clinical trials are urgently needed to confirm whether GLP-1 agonists could be used to treat AUD and SUDs.

## Notes

Supplement 1.
eMethods

eReferences
**eTable 1.** Risk of different outcome events associated with use of medications for alcohol use disorder (AUD) (compared with non-use time of AUD medications) in within-individual model of persons with AUD **eFigure 1.** Principles of how comparisons are conducted in within-individual design where each individual act as his/her own control **eFigure 2.** Encoding of variables, exposures and outcomes **eTable 2.** Sensitivity analysis of the main outcome (AUD hospitalization) restricted with years of market approvals for specific GLP-1 agonists analyzed in within-individual model **eTable 3.** Risk of AUD hospitalization associated with Sodium-glucose linked transporter-2 (SGLT-2) inhibitor use, compared to non-use of SGLT-2 inhibitors in within-individual model **eAppendix.** Study strengths and weaknesses

Supplement 2. Data sharing statement
