# Supplementary material for: Repurposing Semaglutide and Liraglutide for Alcohol Use Disorder
Source: JAMA Psychiatry. 2024 Nov 13;82(1):94–8. doi: 10.1001/jamapsychiatry.2024.3599 (PMC11561716; doi:10.1001/jamapsychiatry.2024.3599)
Supplement: Supplement 1. — eMethods eReferences eTable 1. Risk of different outcome events associated with use of medications for alcohol use disorder (AUD) (compared with non-use time of AUD medications) in within-individual model of persons with AUD eFigure 1. Principles of how comparisons are conducted in within-individual design where each individual act as his/her own control eFigure 2. Encoding of variables, exposures and outcomes eTable 2. Sensitivity analysis of the main outcome (AUD hospitalization) restricted with years of market approvals for specific GLP-1 agonists analyzed in within-individual model eTable 3. Risk of AUD hospitalization associated with Sodium-glucose linked transporter-2 (SGLT-2) inhibitor use, compared to non-use of SGLT-2 inhibitors in within-individual model eAppendix. Study strengths and weaknesses [file jamapsychiatry-e243599-s001.pdf]

Supplemental Online Content

Lähteenvuo M, Tiihonen J, Solismaa A, Tanskanen A, Mittendorfer-Rutz E, Taipale H. Repurposing semaglutide and liraglutide for alcohol use disorder. *JAMA Psychiatry*. Published online November 13, 2024. doi:10.1001/jamapsychiatry.2024.3599

eMethods

eReferences

eTable 1. Risk of different outcome events associated with use of medications for alcohol use disorder (AUD) (compared with non-use time of AUD medications) in within-individual model of persons with AUD

eFigure 1. Principles of how comparisons are conducted in within-individual design where each individual act as his/her own control

eFigure 2. Encoding of variables, exposures and outcomes

eTable 2. Sensitivity analysis of the main outcome (AUD hospitalization) restricted with years of market approvals for specific GLP-1 agonists analyzed in within-individual model

eTable 3. Risk of AUD hospitalization associated with Sodium-glucose linked transporter-2 (SGLT-2) inhibitor use, compared to non-use of SGLT-2 inhibitors in within-individual model

eAppendix. Study strengths and weaknesses

This supplemental material has been provided by the authors to give readers additional information about their work.

© 2024 Lähteenvuo M et al. *JAMA Psychiatry.*

eMethods

Exposure
Exposure was evaluated using the PRE2DUP-method. It uses data from pharmacy dispensings/purchases (dates, amounts and medication type (strength, formulation, etc..)) and turns this data into drug use periods, giving a day-by-day estimate of whether a drug was used or not. The PRE2DUP method is based on the calculation of sliding averages of defined daily dosages, the amounts of drugs purchased, and individual drug use patterns. It takes into account hospital stays and medicine stockpiling. The construction of drug use periods is controlled with clinically meaningful minimum and maximum doses designed for each drug package. It does not use artificial grace periods. It tracks every medication individually and can thus also be used to define polypharmacy[1,2].

Outcome
Outcomes were defined based on primary discharge diagnosis of each hospital care period.

Study design and statistical analysis
Stratified Cox regression analyses were used to conduct within-individual analyses to calculate the risk of an outcome associated to use versus non-use of pharmacotherapies[3]. In within-individual analysis each individual acts as his/her own control, controlling for bias arising from permanent and semi-permanent characteristics (eFigure 1 and 2). The model is suitable for recurring outcomes, but only individuals with variation in both exposure and outcome directly contribute to the estimation of adjusted hazard ratios. In this study, all individuals exposed to GLP-1 agonists were included in the GLP-1 agonist analysis and all individuals exposed to AUD medications were included in analysis of those, regardless of variation in exposure or having the outcome event (they contributed to analysis indirectly, via time-varying covariates).

Additional analyses were done to control for bias arising from the different market entry times of the medications. In the first analysis, the start of follow-up was defined according to market entry as January 1st 2009 for liraglutide, January 1st 2014 for dulaglutide and January 1st 2018 for semaglutide (eTable 2). In the second analysis, a negative control not likely to affect risk of AUD hospitalization, but with a similar market entry time as for the GLP-1 agonists, was used (eTable 3). For this analysis Sodium-glucose linked transporter-2 (SGLT-2) inhibitors were used as negative controls and start of follow-up was restricted to start when each drug received approval from European Medicines Agency, namely 2012 for dapagliflozin and 2014 for empagliflozin.

The models were adjusted for time-varying use of psychotropic medications (antipsychotics N05A, antidepressants N06A, mood stabilizers including carbamazepine N03AF01, valproic acid N03AG01, lamotrigine N03AX09 and lithium N05AN01), benzodiazepines and related drugs (N05BA, N05CD, N05CF), ADHD medications (N06BA) and use of other antidiabetic drugs than GLP-1 agonists (A10 excluding A10BJ). In addition, the analyses were adjusted for temporal order of GLP-1 agonists and time since cohort entry.

eReferences

1. Tanskanen A, Taipale H, Koponen M, et al. From prescription drug purchases to drug use periods - A second generation method (PRE2DUP). *BMC Med Inform Decis Mak*. 2015;15(1). doi:10.1186/s12911-015-0140-z

2. Taipale H, Tanskanen A, Koponen M, Tolppanen AM, Tiihonen J, Hartikainen S. Agreement between PRE2DUP register data modeling method and comprehensive drug use interview among older persons. *Clin Epidemiol*. 2016;8:363-371. doi:10.2147/CLEP.S116160

3. Lichtenstein P, Halldner L, Zetterqvist J, et al. Medication for Attention Deficit–Hyperactivity Disorder and Criminality. *New England Journal of Medicine*. 2012;367(21):2006-2014. doi:10.1056/nejmoa1203241

© 2024 Lähteenvuo M et al. *JAMA Psychiatry.*

eTable 1. **Risk of different outcome events associated with use of medications for alcohol use disorder (AUD) (compared with non-use time of AUD medications) in within-individual model of persons with AUD** (adjusted for time-varying use of psychotropic medications (antipsychotics N05A, antidepressants N06A, mood stabilizers including carbamazepine N03AF01, valproic acid N03AG01, lamotrigine N03AX09 and lithium N05AN01), benzodiazepines and related drugs (N05BA, N05CD, N05CF), ADHD medications (N06BA) and use of other antidiabetic drugs than GLP-1 agonists (A10 excluding A10BJ), temporal order of AUD medication and time since cohort entry).

	Events	Users	PYs	aHR (95% CI)
AUD hospitalization				
Disulfiram	12652	44264	33134	0.98 (0.96-1.00)
Acamprosate	9061	31555	17575	1.11 (1.08-1.15)
Naltrexone	4772	31660	15032	0.86 (0.83-0.89)
SUD hospitalization				
Disulfiram	5673	44264	33134	0.98 (0.96-1.00)
Acamprosate	6250	31555	17575	1.12 (1.09-1.15)
Naltrexone	481	31660	15032	0.86 (0.84-0.90)
Somatic hospitalization				
Disulfiram	50	44264	33134	0.84 (0.81-0.88)
Acamprosate	613	31555	17575	0.94 (0.90-0.99)
Naltrexone	362	31660	15032	0.78 (0.73-0.84)
Suicide attempt				
Disulfiram	1636	44264	33134	1.21 (1.12-1.30)
Acamprosate	699	31555	17575	1.25 (1.10-1.41)
Naltrexone	522	31660	15032	0.95 (0.84-1.09)

© 2024 Lähteenvuo M et al. *JAMA Psychiatry.*

eFigure 1. Principles of how comparisons are conducted in within-individual design where each individual act as his/her own control

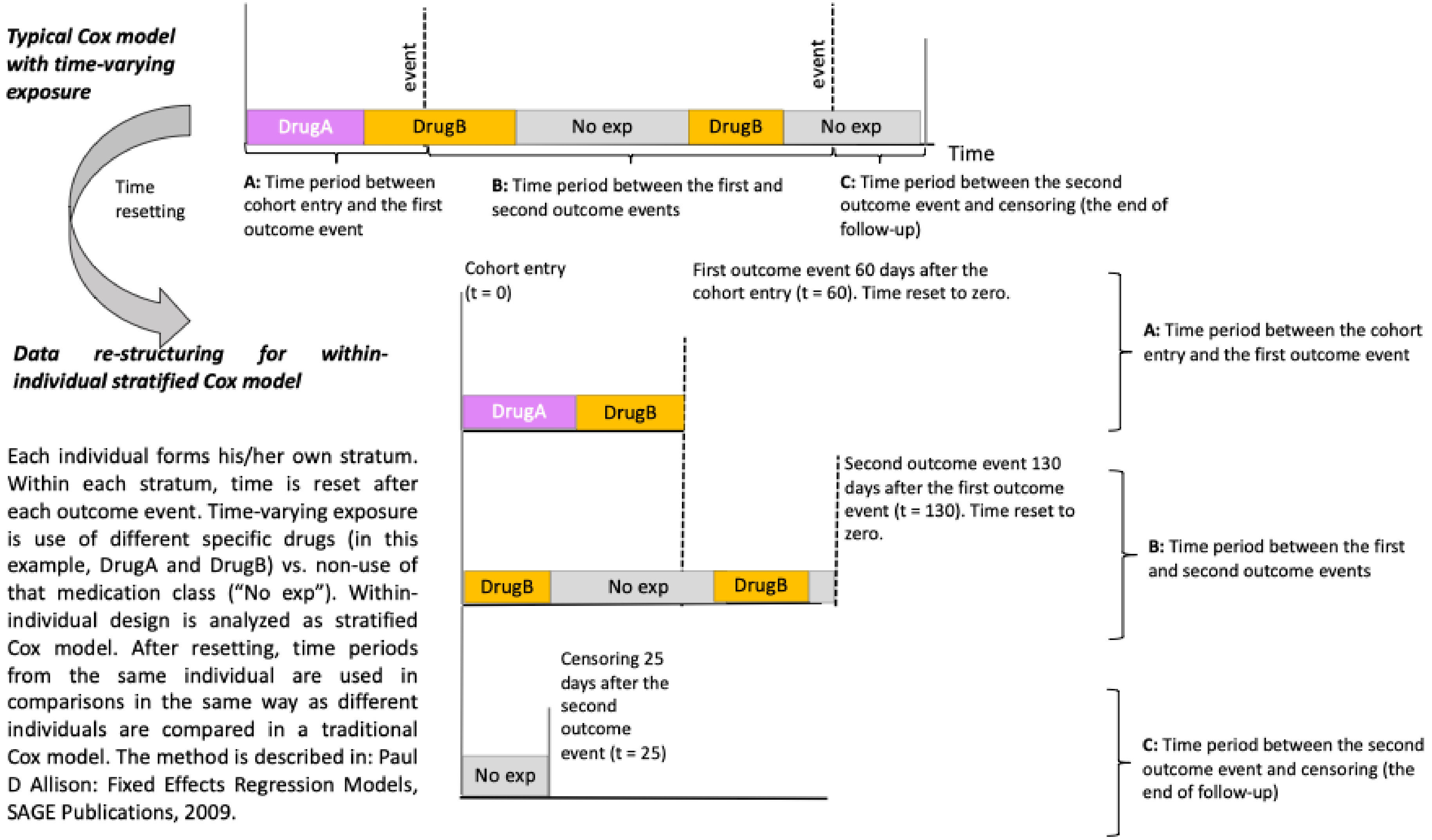

© 2024 Lähteenvuo M et al. *JAMA Psychiatry.*

eFigure 2. Encoding of variables, exposures and outcomes.

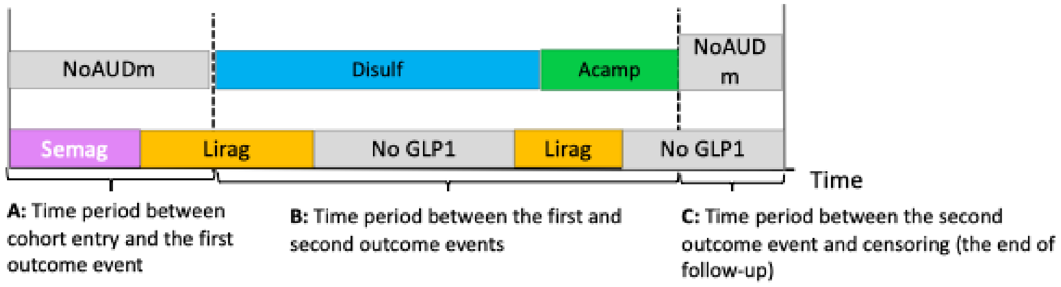

Structure of the analyses dataset and variables for the example drug use history above

row#	ID	start	stop	GLP	AUD	AD	BE	series	censor	init	end
1	3	1	29	Semag	NoAUDm	1	0	1	0	1	29
2	3	30	60	Lirag	NoAUDm	1	0	1	1	30	60
3	3	61	92	Lirag	Disulf	1	0	2	0	1	32
4	3	93	98	NoGLP1	Disulf	1	0	2	0	33	38
5	3	99	130	NoGLP1	Disulf	0	0	2	0	39	70
6	3	131	140	NoGLP1	Disulf	0	0	2	0	71	80
7	3	141	146	Lirag	Disulf	0	0	2	0	81	109
8	3	147	180	Lirag	Acamp	0	1	2	0	109	120
9	3	181	190	NoGLP1	Acamp	0	1	2	1	121	130
10	3	191	215	NoGLP1	NoAUDm	0	1	3	0	1	25

start and stop describe continuous time since cohort entry (start=1) until the end of follow-up (stop=215) as days. These are the basis for between-individual model.
GLP describes time-varying use of two specific GLP-1 agonists (named “Lirag” and “Semag”) and non-use of GLP-1 agonists (“No GLP1”).
AUD describes the use (“Disulf” and “Acamp”) versus non-use of AUD medications (“No AUDm”).
Variables AD and BE describe time-varying use of antidepressants and benzodiazepines for which analyses were adjusted for (1=use, 0=non-use). In this study, analysis were also adjusted for the use of antipsychotics, mood stabilizers and ADHD medications which were coded similarly as AD and BE (left out for simplicity, not shown in figure).
series indicates which rows belong to the same series of within-individual model (when series changes time is reset to zero).
censor indicates whether the period ended at outcome event (censor=1) or censoring (censor=0). After censor=1, time is reset and the next row starts with init=1.
init and end assign time variable for within-individual model where time is reset to zero after each outcome event.

© 2024 Lähteenvuo M et al. *JAMA Psychiatry.*

eTable 2. Sensitivity analysis of the main outcome (AUD hospitalization) restricted with years of market approvals for specific GLP-1 agonists analyzed in within-individual model. Start of follow-up was defined according to market entry as January 1st 2009 for liraglutide, January 1st 2014 for dulaglutide and January 1st 2018 for semaglutide.

	Events	Users	PYs	aHR (95%CI)
Liraglutide since 2009				
Non-use of GLP-1 agonists	9367	6252	58462	
Liraglutide	212	2509	3076	0.73 (0.58-0.92)
Dulaglutide since 2014				
Non-use of GLP-1 agonists	4872	6234	42468	
Dulaglutide	139	1118	1443	0.95 (0.67-1.33)
Semaglutide since 2018				
Non-use of GLP-1 agonists	2572	6142	25618	
Semaglutide	222	4321	4677	0.68 (0.51-0.89)

eTable 3. Risk of AUD hospitalization associated with Sodium-glucose linked transporter-2 (SGLT-2) inhibitor use, compared to non-use of SGLT-2 inhibitors in within-individual model.

	Events	Users	PYs	aHR (95%CI)	Time-restricted aHR (95%CI)*
Non-use of SGLT-2 inhibitors	13009	6970	67164	reference	reference
Dapagliflozin	414	2838	3555	1.00 (0.76-1.33)	1.07 (0.80-1.42)
Empagliflozin	630	4452	7961	0.84 (0.64-1.09)	0.88 (0.67-1.16)

*Start of follow-up restricted by time when each drug received approval from European Medicines Agency, namely 2012 for dapagliflozin and 2014 for empagliflozin.

eAppendix

Study strengths and weaknesses

The strengths of this study include a large and inclusive cohort analyzed with validated methods. However, as this is an observational study, it can only speak for associations, not causality, and possible mechanisms behind the associations are beyond reach for this study. The within-individual analysis used eliminates bias arising from permanent or semi-permanent characteristics. Some forms of biases still remain. Firstly, we could not ascertain for what diagnosis the GLP-1 agonists were initially prescribed for. They may have different effects for individuals suffering from diabetes or obesity or both. This study was not able to obtain data on amount or activity of substance use, and thus all outcomes used are merely proxies, although do represent very meaningful and concrete consequences. Also, this study could not account for benefits/problems not showing up in registries, such as a decrease in amounts consumed or improvement in quality of life or daily functioning. Also, SUDs may have different stages. The effect of medications may be different whether they are started during periods of abstinence or active use, but this study was unable to control for this effect. Also, the analysis assumes that changes in exposure should not be dependent on outcomes. This is likely true for the analyses for GLP-1 agonists, but hospitalizations due to AUD may have modified the risk of a patient receiving an AUD medication prescription, which may have introduced some bias to the results presented for AUD-medications, possibly leading to the underestimation of their effectiveness. Another underlying assumption of the model is that outcomes are independent. It is possible that being hospitalized for an AUD can affect the willingness of a patient to seek further hospitalizations in the future, either reducing or increasing this willingness, which may also have led to bias.

© 2024 Lähteenvuo M et al. *JAMA Psychiatry.*
