# Supplementary material for: Repurposing Semaglutide and Liraglutide for Alcohol Use Disorder
Source: JAMA Psychiatry. 2024 Nov 13;82(1):94–8. doi: 10.1001/jamapsychiatry.2024.3599 (PMC11561716; doi:10.1001/jamapsychiatry.2024.3599)
Supplement: Supplement 2. — Data sharing statement [file jamapsychiatry-e243599-s002.pdf]

Data Sharing Statement

Lähteenvuo. Repurposing Semaglutide and Liraglutide for Alcohol Use Disorder. *JAMA Psychiatry*. Published November 13, 2024. doi:10.1001/jamapsychiatry.2024.3599

Data
Data available: No

Additional Information
Explanation for why data not available: The data used in this study cannot be made publicly available due to privacy regulations. According to the General Data Protection Regulation, the Swedish law SFS 2018:218, the Swedish Data Protection Act, the Swedish Ethical Review Act, and the Public Access to Information and Secrecy Act, these types of sensitive data can only be made available for specific purposes, including research, that meets the criteria for access to this sort of sensitive and confidential data as determined by a legal review. Readers may contact Professor Kristina Alexanderson (kristina.alexanderson@ki.se) regarding the data.
